# How do compression and flexion-compression injuries destabilize the spine? A novel *in vitro* protocol for analyzing three-dimensional biomechanical instability

**DOI:** 10.3389/fbioe.2025.1576720

**Published:** 2025-05-19

**Authors:** Ann-Kathrin Greiner-Perth, Hans-Joachim Wilke, Christian Liebsch

**Affiliations:** Institute of Orthopaedic Research and Biomechanics, Trauma Research Centre Ulm, Ulm University, Ulm, Germany

**Keywords:** trauma, injury, thoracic spine, compression, flexion-compression, vertebral fracture, biomechanical instability, *in vitro* study

## Abstract

**Introduction:**

Unstable traumatic spinal injuries require surgical stabilization. However, biomechanical instability of specific spinal injuries has been little investigated, although restoring stability represents a primary goal of surgical treatment. This study aimed (1) to develop an *in vitro* protocol to generate standardized spinal compression injuries, (2) to establish a three-dimensional flexibility analysis to identify relevant biomechanical instability parameters, and (3) to examine effects of person-specific factors on vertebral fragility.

**Methods:**

Mechanical fracture simulation was performed on twelve fresh-frozen human spine specimens (T9-11; 4 f/8 m; 40–60 years) using a material testing machine. Pure compression trauma (n = 6) was simulated by applying displacement-controlled axial compression at 300 mm/s until 20% compression of the T10 vertebral body height. Flexion-compression trauma (n = 6) was achieved by additional flexural loading of 10 Nm. Pre- and post-traumatic pure moment testing with 5 Nm was performed in flexion/extension, lateral bending, and axial rotation using optical motion tracking to determine range of motion (ROM), neutral zone (NZ), coupled rotations, and coupled translations. Translations under shear loading of 100 N and axial deformation under 400 N compression were analyzed.

**Results:**

All specimens exhibited AOSpine A1 injuries occurring at a median fracture load of 5.0 kN (2.4–9.2 kN). Pure compression generated isolated medial endplate fractures (n = 5), while flexion-compression primarily provoked combined endplate and ventral compression injuries (n = 3). Significant (p < 0.05) increases were detected for all parameters except for coupled rotations and posterior (compression) and left shear translation (flexion-compression). Highest instability increases were determined for axial deformability (compression: +136% / flexion-compression: +200%) and NZ (flexion/extension: +177% / 188%; lateral bending: +174% / +126%). Mild to moderate disc degeneration and age did not correlate with fracture loads (p > 0.05). In compression trauma, cortical bone mineral density (BMD) of T10 had no effect on fracture loads (p > 0.05), whereas in flexion-compression trauma, a significant (p < 0.05) linear correlation was found (Spearman’s r_s_ = 0.83).

**Discussion:**

Relevant instability parameters of minor compression and flexion-compression injuries include axial deformability, NZ, ROM, and coupled translations. Cortical BMD of the target vertebra solely affects fracture generation in flexion-compression trauma. Consequently, risk factors for fracture development may vary between trauma mechanisms.

## 1 Introduction

Traumatic injuries of the spine represent a substantial public health issue, given the considerable prevalence of associated morbidity and mortality rates (Hasler et al., 2011; Wood et al., 2014). Vertebral fractures and dislocations often result in poor functional outcomes, significantly reduced quality of life, and a low rate of return to work (Bouyer et al., 2015). Most often, spinal trauma is a consequence of serious traumatic events, including falls, motor vehicle, traffic, or sports accidents (Lenehan et al., 2009; Leucht et al., 2009; Niemi-Nikkola et al., 2018; Tafida et al., 2018; den Ouden et al., 2019). Among adult trauma patients, the global incidence of spinal trauma has been reported to vary between 20 and 70 cases per 100,000 people per year (Hu et al., 1996; Roche et al., 2008; Liu et al., 2018; Niemi-Nikkola et al., 2018), with the majority occurring in patients under the age of 60 years (Lenehan et al., 2009; Hasler et al., 2011; Tafida et al., 2018). Approximately 10% of trauma patients experience isolated spinal fractures or dislocations without accompanying neurological deficits (Hasler et al., 2011). The thoracolumbar junction (T12-L1: 45%) represents the most prevalent site of traumatic thoracolumbar injury, yet significant incidences are also observed in the thoracic (T1-T11: 27%) and lumbar spine (L2-L5: 28%) (Magerl et al., 1994).

Given the high clinical relevance, optimal management of traumatic spinal injuries is of great importance. However, the choice of the optimal treatment strategy for traumatic thoracolumbar fractures remains one of the most controversial topics in the field of spinal traumatology. According to currently available treatment guidelines, unstable injuries and injuries associated with neurological deficits require surgical treatment to stabilize the fracture, to correct post-traumatic kyphosis, to decompress the spinal canal and to prevent further neurological injury (Verheyden et al., 2018). Nevertheless, literature reports a complication rate of approximately 40% for surgically treated severe thoracolumbar injuries (Cabrera et al., 2023; Adegeest et al., 2025). To standardize epidemiological research and to provide guidance in clinical practice, several classification systems have been introduced to categorize spinal injury patterns (Denis, 1983; Magerl et al., 1994; Vaccaro et al., 2013). One of the most widely accepted classification systems for thoracolumbar injuries represents the AOSpine classification developed by Schnake et al. (2017), which is based on the classification scheme by Magerl et al. (1994). The AOSpine classification system distinguishes three main categories based on injury morphology, trauma mechanism, and clinical factors such as presence of neurological deficits: spinal compression injuries (AOSpine type A), ligamentous injuries (AOSpine type B), and rotational or translational injuries (AOSpine type C). However, biomechanical instability criteria are not yet included in this classification.

Particularly with regard to unstable injuries, there is a lack of information about which type of spinal injury causes which degree of instability and what surgical approach is best to restore spinal stability (Liebsch and Wilke, 2022c). This may be due to the fact that currently no practical definition of spinal instability exists. White and Panjabi were first to define clinical instability in more theoretical terms as “the loss of the ability of the spine under physiologic loads to maintain its pattern of displacement so that there is no initial or additional neurologic deficit, no major deformity, and no incapacitating pain” (White and Panjabi, 1990). Subsequently, various authors have defined spinal instability indirectly based on radiological, morphological, or clinical criteria or attributed instability to fracture mechanisms (Abbasi Fard et al., 2017). A more precise definition on a biomechanical basis is currently not available. One reason for this may be that direct clinical measurement of biomechanical spinal instability is not feasible due to the safety risks when applying loads to patients with potentially unstable fractures. Consequently, biomechanical assessment of effects of traumatic injuries on altered spinal flexibility has so far relied on *in vitro* testing using injury models.

One of the first *in vitro* injury models for thoracolumbar burst fractures was introduced by Willen et al. (1984) who employed a drop tower to simulate a single compression trauma. However, determining the precise load required to generate injuries of specific severity remained a significant challenge. Panjabi et al. developed an incremental trauma protocol using a drop tower to induce defined fracture morphologies in the thoracolumbar spine by using gradually increasing drop weights (Panjabi et al., 1994b; Panjabi et al., 2000). This approach was limited by the fact that no clinically representative fracture evolution was simulated due to the incremental nature of fracture progression. Subsequently, other research groups developed various protocols for simulating compression injuries in the thoracolumbar and lumbar spine, again using mechanical injury simulations with drop towers (Ching et al., 1995; Kifune et al., 1995; Wang et al., 2007; Jones et al., 2011; Brandolini et al., 2014), material testing machines (Shirado et al., 1992; Ching et al., 1995), or manual standardized defect creation by resection, while others combined mechanical and manual injury simulation (Hartensuer et al., 2012; Oberkircher et al., 2016; Germaneau et al., 2017). In terms of physiological fracture development and clinically relevant injury morphologies, it has been shown that purely mechanical trauma simulation appears to provide the greatest comparability with clinical injury patterns (Liebsch and Wilke, 2022c).

Using these *in vitro* injury models, numerous research groups have investigated altered spinal flexibility after trauma (Liebsch and Wilke, 2022c). Biomechanical investigations of spinal flexibility usually require the use of quantitative measures to verify changes in flexibility and to ensure adequate validity of the results. In terms of instability analysis, spinal instability can therefore be characterized by exceeding physiological biomechanical parameter thresholds. However, there is currently no consensus on whether increased flexibility alone already indicates spinal instability. Nonetheless, since *in vitro* injury models do not account for the effect of spinal trauma on neurology or overall stability, considering also active muscle stabilization, any increase in biomechanical flexibility beyond that of an intact spine can therefore be considered as biomechanically unstable. Analysis of spinal flexibility involves determining biomechanical parameters, such as range of motion, neutral zone, coupled motions, and translational flexibility. While range of motion (ROM) is defined as the maximum extent to which one or more spinal motion segments can deform under physiological loading, neutral zone (NZ) characterizes spinal laxity in the neutral position in an unloaded state. In addition to primary motion analysis, coupled rotations describe rotational motion components in secondary motion planes due to bending loads. Translational flexibility refers to the relative linear horizontal motion of a spinal motion segment and can be further differentiated into coupled and shear translation. Coupled translation describes relative linear deformation during primary rotational motion, while shear translation is induced by antero-posterior and latero-lateral shear loading. Despite the numerous parameters describing spinal flexibility, the majority of biomechanical studies have primarily focused on analyzing changes in range of motion, while only a few have additionally examined the neutral zone (Liebsch and Wilke, 2022c). Therefore, a comprehensive understanding of biomechanical spinal instability associated with specific injury types, particularly in the thoracic spine, that goes beyond the assessment of altered range of motion and neutral zone, is still lacking, as investigations into the effects of spinal trauma on other biomechanical parameters are still scarce (Liebsch and Wilke, 2022c).

The purpose of this *in vitro* study therefore was to develop a standardized injury model based on a purely mechanical trauma simulation that replicates pure compression or flexion-compression trauma and to perform a detailed multiparametric instability analysis to determine biomechanical spinal instability following trauma. Additionally, this study sought to examine the impact of person-specific factors on vertebral fragility and fracture development, with the aim of enhancing the understanding of underlying traumatic injury mechanisms.

## 2 Materials and methods

### 2.1 Specimens

Twelve fresh-frozen bisegmental thoracic spinal specimens (T9-T11) were gathered from human donors for biomechanical testing. Approval for the usage of human specimens was provided by the ethical committee board of the University of Ulm, Germany (no. 407/21). The specimens were obtained from an ethically approved body donation program (Science Care Inc., Phoenix, Arizona, USA). Four specimens originated from female and eight from male donors. The median age of the donor group was 53 years, ranging from 40 to 60 years (Table 1). Prior to testing, the specimens were analyzed by computed tomography (CT, Siemens Somatom Definition AS, Siemens Healthcare, Erlangen, Germany) to exclude previous vertebral fractures, spinal deformities, and severe degeneration. Quantitative computed tomography was used to ascertain trabecular and cortical bone mineral density (BMD) of the T10 vertebra, which was selected for fracture in each specimen (Table 1). However, BMD data were absent for one specimen, which was consequently excluded from correlation analyses pertaining to BMD in the T10 vertebral body. In order to divide all specimens into two comparable groups based on BMD, the BMD values of the adjacent vertebrae were considered for this particular specimen. Mean BMD of all T10 vertebrae examined was 96 mgCA-HA/ml, ranging from 59 to 152 mgCA-HA/ml.

**TABLE 1:** Donor and specimen specific data and fracture loads, fracture morphology and fracture classification post-trauma.

Testing group	Donor no.	Sex	Age	Segment	Degree of disc degeneration (Liebsch et al., 2022)	Trabecular BMD of T10 in mg CA-HA/ml	Cortical BMD of T10 in mg CA-HA/ml	Anterior VBH of T10 in mm	Fracture load in kN	Fracture morphology	Schnake/AO (Schnake et al., 2017)
Group 1: Compression trauma	1	Male	49	T9-T11	T9-T10: 2 T10-T11: 2	128	168	21.3	6.42	Endplate fracture	A1
	2	Female	48	T9-T11	T9-T10: 1 T10-T11: 1	123	160	22.7	3.53	Endplate fracture	A1
	3	Female	56	T9-T11	T9-T10: 1 T10-T11: 1	101	175	20.9	4.14	Endplate fracture	A1
	4	Male	53	T9-T11	T9-T10: 2 T10-T11: 2	-	-	22.6	7.16	Endplate fracture	A1
	5	Male	55	T9-T11	T9-T10: 2 T10-T11: 2	83	172	16.8	5.16	Compression injury	A1
	6	Male	47	T9-T11	T9-T10: 2 T10-T11: 2	59	238	22.8	4.81	Endplate fracture	A1
Median (Min, Max)			51 (47,56)			101 (59, 128)	172 (160, 238)	21.9 (16.8, 22.8)	4.99 (3.53, 7.16)		
Group 2: Flexion-compression trauma	7	Male	58	T9-T11	T9-T10: 2 T10-T11: 2	152	394	22.3	9.21	Endplate fracture	A1
	8	Male	49	T9-T11	T9-T10: 2 T10-T11: 1	123	286	20.0	7.92	Compression injury + Endplate fracture	A1
	9	Male	58	T9-T11	T9-T10: 1 T10-T11: 1	96	244	20.9	4.15	Compression injury	A1
	10	Male	40	T9-T11	T9-T10: 2 T10-T11: 1	83	250	21.8	5.42	Compression injury + Endplate fracture	A1
	11	Female	59	T9-T11	T9-T10: 2 T10-T11: 1	70	142	19.8	2.39	Compression injury + Endplate fracture	A1
	12	Female	60	T9-T11	T9-T10: 2 T10-T11: 2	65	126	20.0	4.86	Compression injury	A1
Median (Min, Max)			58 (40,60)			90 (65, 152)	247 (126, 394)	20.4 (19.8, 22.3)	5.14 (2.39, 9.21)		

Min, Minimum; Max, Maximum; BMD, bone mineral density; VBH, vertebral body height.

The specimens were stored at −20°C and thawed overnight for at least 12 h at 4°C prior to preparation and testing. During preparation, muscle and soft tissues were dissected while preserving bony and ligamentous structures including costovertebral and costotransverse joints at the T9-T10 level. After preparation, the T9 and T11 vertebrae were completely embedded in PMMA (Technovit 3040, Heraeus Kulzer, Wehrheim, Germany), leaving the adjacent intervertebral discs free to allow full mobility while providing maximum stabilization of the cranial and caudal vertebrae and horizontal alignment of the embedding to the endplates of the T10 vertebra. For biomechanical testing, the PMMA blocks were equipped with flanges for mounting in the testing machines and three retroreflective markers for motion analysis. 0.9% saline solution was used to keep the specimens moist during testing according to established testing criteria for human spinal specimens (Wilke et al., 1998) in order to maintain physiological motion characteristics of the intervertebral discs and ligaments.

### 2.2 Morphological analysis

Prior to trauma simulation, person-specific criteria were assessed. The degree of disc degeneration at the T9-T10 and T10-T11 intervertebral discs was determined using a grading system for thoracic disc degeneration (Liebsch et al., 2022) based on pre-trauma radiographs taken with a full-guard radiographical unit (Faxitron 43805N, Hewlett-Packard, Palo Alto, USA). Morphological parameters were evaluated based on DICOM data acquired by CT scans prior to trauma (CT, Siemens Somatom Definition AS, Siemens Healthcare, Erlangen, Germany) using an open-source medical image viewer (MicroDicom DICOM Viewer, MicroDicom Ltd., Sofia, Bulgaria):

• Vertebral body height (VBH): For each vertebra, VBH was measured orthogonally from the cranial to the caudal endplate. VBH was determined anteriorly (aVBH), medially (mVBH), and posteriorly (pVBH).
• Intervertebral disc height (IVDH): IVDH of T9-T10 and T10-T11 intervertebral discs was determined by evaluating the orthogonal distance between the adjacent cranial and caudal endplates. IVDH was analyzed anteriorly (aIVDH), medially (mIVDH), and posteriorly (pIVDH).
• Cross-sectional area (CSA): In addition, CSA of the target vertebral body T10 was measured at mid-height of the vertebral body and at the level of the cranial and caudal endplates.

### 2.3 Trauma simulation

**FIGURE 1:**
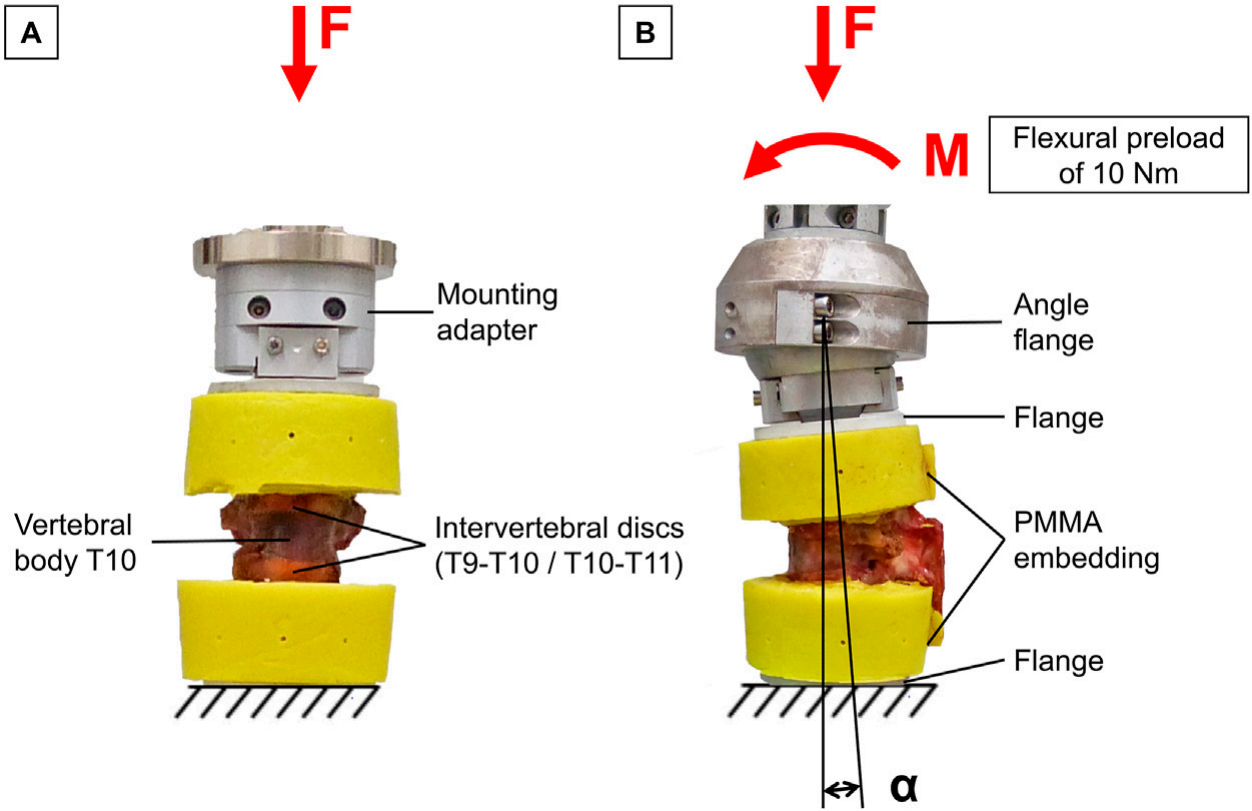
Experimental trauma simulation: **(A)** Pure compressive loading of thoracic bisegmental specimens (T9-T11) to simulate pure compression trauma. **(B)** Additional flexural preload applied on bisegmental specimens (T9-T11) followed by axial compressive impact to simulate flexion-compression trauma.

For trauma simulation, the specimens were subjected to a dynamic, displacement-controlled impact using a material testing machine (Instron 8871, Instron Corporation, MA, USA). The specimens were preconditioned by applying an axial preload of 400 N for 10 s simulating body weight acting on the thoracic spine (Anderson et al., 2016). The twelve specimens were then divided into two groups of six specimens based on the bone mineral density of the T10 vertebra, ensuring that each group included healthy (≥120 mgCA-HA/ml), osteopenic (120–80 mgCA-HA/ml), and osteoporotic (≤80 mgCA-HA/ml) specimens. Specimens of the first group were subjected to dynamic axial compression loading with a target velocity of 300 mm/s up to a compression of 20% of the anterior vertebral body height of T10 according to Hartensuer et al. (Hartensuer et al., 2012), resulting in pure compression trauma (Figure 1A). The specimens of the second study group were additionally loaded with a static flexural preload of 10 Nm and subsequently subjected to the above-described dynamic axial compression impact, resulting in flexion-compression trauma (Figure 1B). The target vertebral body of each specimen was centrally aligned in both the sagittal and frontal planes to ensure central load application in both trauma groups. Data acquisition during trauma simulations was performed at a sampling rate of 1 kHz. After trauma simulation, the occurrence of traumatic injuries was assessed for each specimen. Traumatic injury was defined as a load drop of at least 10% during trauma application. The maximum load before the first load drop was specified as fracture load. For fracture detection, radiographic examination was performed post-trauma using a mobile x-ray unit (Mobilett XP, Siemens Healthcare GmbH, Erlangen, Germany). Radiographs of the tested specimens were taken both in their unloaded state and under an axial load of 400 N. For injury classification, the specimens were subjected to additional CT scans (CT, Siemens Somatom Definition AS, Siemens Healthcare, Erlangen, Germany). Based on these radiographs and CT images, the simulated injuries were classified according to the AOSpine classification system for thoracolumbar injuries by Schnake et al. (2017).

### 2.4 Multiparametric instability analysis

Before and after trauma simulation, a detailed multiparametric flexibility analysis was performed to detect increases in flexibility, i.e., instability, following trauma application.

#### 2.4.1 Flexibility analysis

**FIGURE 2:**
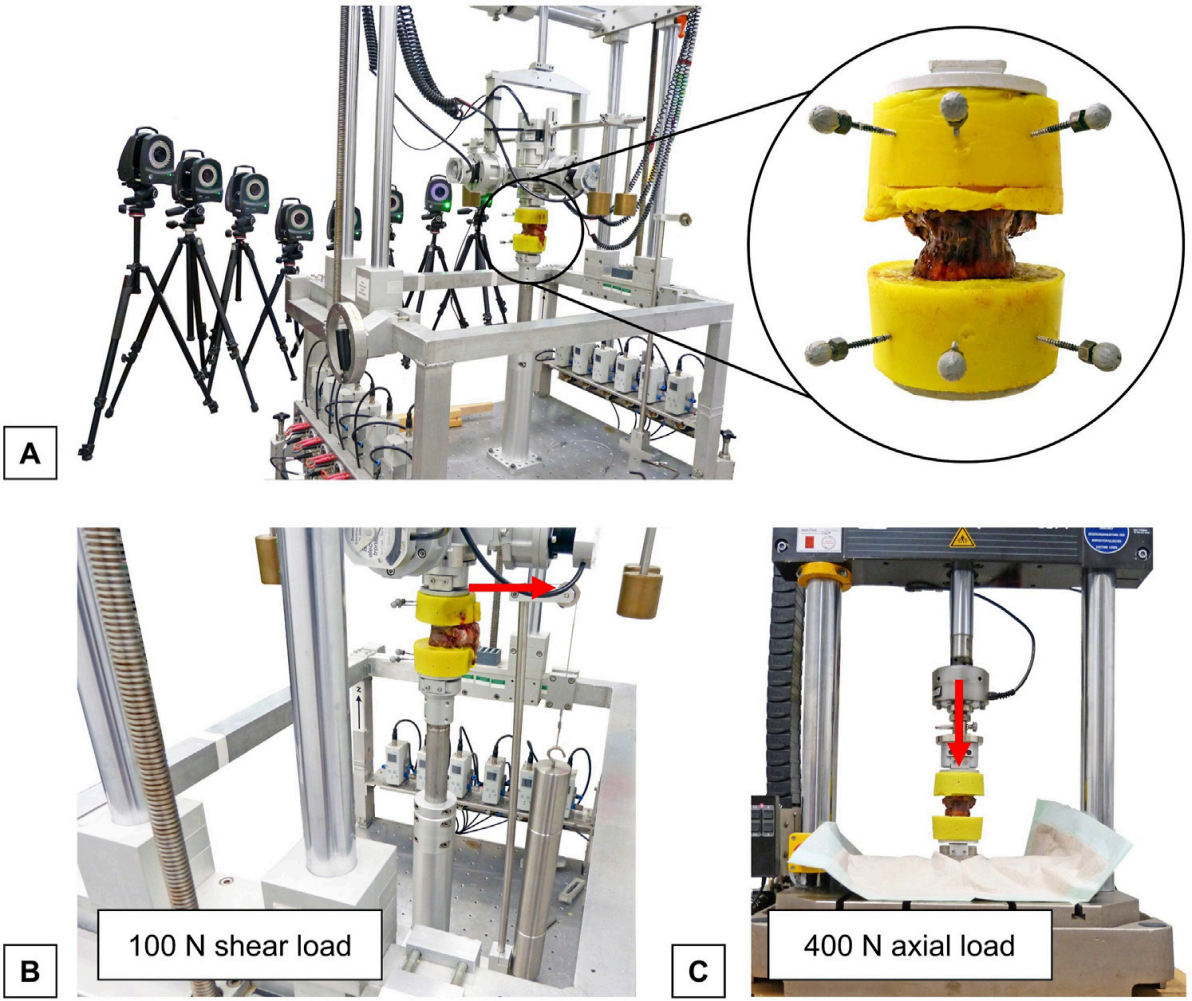
Experimental setup for flexibility testing: **(A)** Setup for flexibility testing showing a thoracic bisegmental specimen (T9-T10) with three retroreflective markers fixed to each embedding in the universal spine tester (Wilke et al., 1994) and the optical motion tracking system consisting of eight cameras (Vicon Motion Systems Ltd., Oxford, UK). **(B)** Setup for the analysis of shear translatory flexibility showing the thoracic specimen loaded with a posterior shear load (red arrow) in the spine tester. **(C)** Setup for the analysis of axial deformability showing specimen in a material testing machine (Instron 8871, Instron Corporation, MA, USA) under axial loading (red arrow).

Quasi-static flexibility testing of the specimens was performed using a custom-built, well-established spine tester consisting of a traveling gantry and counterbalance weights, which allow unrestricted movement of the specimens to ensure pure moment loading (Wilke et al., 1994) (Figure 2A). Pure moments of 5 Nm were applied to the specimens in the primary motion planes flexion/extension, lateral bending, and axial rotation under displacement control at an angular velocity of 1°/s. In each motion plane, the load was applied for 3.5 cycles to reduce viscoelastic effects, of which the third full cycle was used for data analysis (Wilke et al., 1998). Prior to testing, the six-component load cell, with a measurement resolution of 0.02 N, was calibrated to ensure a maximum error of <1%. During loading, motion analysis was performed using the Vicon MX13 optical motion tracking system (Vicon Motion Systems Ltd., Oxford, UK) to determine the relative motions of the upper and lower vertebrae. For this purpose, the embedded cranial and caudal vertebrae were each equipped with three optical markers. The markers were captured by eight infrared cameras ventrally positioned around the specimen. Preliminary in-house investigations demonstrated that the optical motion tracking system achieved measurement accuracies far below 0.1 mm and 0.1°. For data synchronization, motion tracking was triggered by the stepping motor signal of the spine tester and motion data was recorded with the same measurement frequency of 50 Hz. By means of the three markers on the cranial and caudal embeddings, local coordinate systems were generated for the respective T9 and T11 vertebrae, allowing evaluation of motions within the motion plane (primary motion) and outside the motion plane (secondary / coupled motions). For data analysis, motion data from the motion tracking system and load data recorded by the spine tester were merged using Matlab (MathWorks Inc., Natick, USA). To determine the primary range of motion (ROM) and neutral zone (NZ), the displacement of each primary motion plane was plotted against the moment of the primary motion direction to create hysteresis curves. Based on the generated hysteresis curve of the third full-load cycle, ROM and NZ of the bisegmental specimens (T9-T11) were analyzed using Matlab.

#### 2.4.2 Coupled motion analysis

In addition to primary ROM and NZ, coupled motions were evaluated. Coupled rotations were evaluated in relation to the load in the primary motion direction by determining the angular displacement in secondary motion direction at maximum load in the primary motion plane. In addition to coupled rotations, coupled translations were analyzed using a custom Python script by merging displacement data in all motion planes and the load data in the primary motion plane. Corresponding to coupled rotations, coupled translations were determined at maximum load for each primary motion, i.e., flexion/extension, lateral bending, and axial rotation. Translations of the cranial vertebral body were evaluated along each spatial axis relative to the caudal vertebral body, which was rigidly fixed in the spine tester, to determine total relative translations.

#### 2.4.3 Translational flexibility analysis

Horizontal translational flexibility due to shear loading was examined in the universal spine tester (Figure 2B). To evaluate horizontal translations, all three rotational degrees of freedom were blocked, while all three translational degrees of freedom remained free within the testing machine. Subsequently, static shear load of 100 N was applied to the cranial vertebrae in the anterior, posterior, and both lateral directions. Relative horizontal displacement was then determined by calculating the difference in displacement in each horizontal load direction between the unloaded and loaded positions using the data obtained from the optical motion analysis system.

#### 2.4.4 Axial deformability analysis

Axial deformation of the specimens was measured under static axial compression (Willen et al., 1984). For this, the specimens were axially loaded with 400 N compressive force using a material testing machine (Instron 8871, Instron Corporation, MA, USA) to simulate body weight loading (Figure 2C). During loading and unloading of the specimens, axial height was measured continuously. Relative axial deformability, termed relative height loss, was then evaluated. For this purpose, the difference in axial deformation between the unloaded and loaded states at 400 N was calculated for each specimen.

### 2.5 Statistics

The multiparametric flexibility data were post-processed using Microsoft Excel (Microsoft Corp., Redmond, USA). To analyze increased flexibility, i.e., instability, after trauma simulation, data of all primary and secondary flexibility parameters, as well as data of horizontal translations and relative height loss, were divided into groups before and after trauma for compression and flexion-compression trauma, respectively. Statistical analysis was performed using the Friedman test in SPSS 24 (IBM Corp., Armonk, NY, USA). The significance level was set to 0.05. Grouped data was visualized as medians with value ranges.

In addition to the instability analysis, correlations between person-specific parameters and vertebral body fragility were evaluated. For each trauma group, effects of age, donor sex, degree of disc degeneration, bone mineral density, and morphological parameters on the fracture load were analyzed. The correlation analysis was also performed in SPSS by calculating Spearman correlation coefficients with a significance level of 0.05.

## 3 Results

### 3.1 Fracture types

**FIGURE 3:**
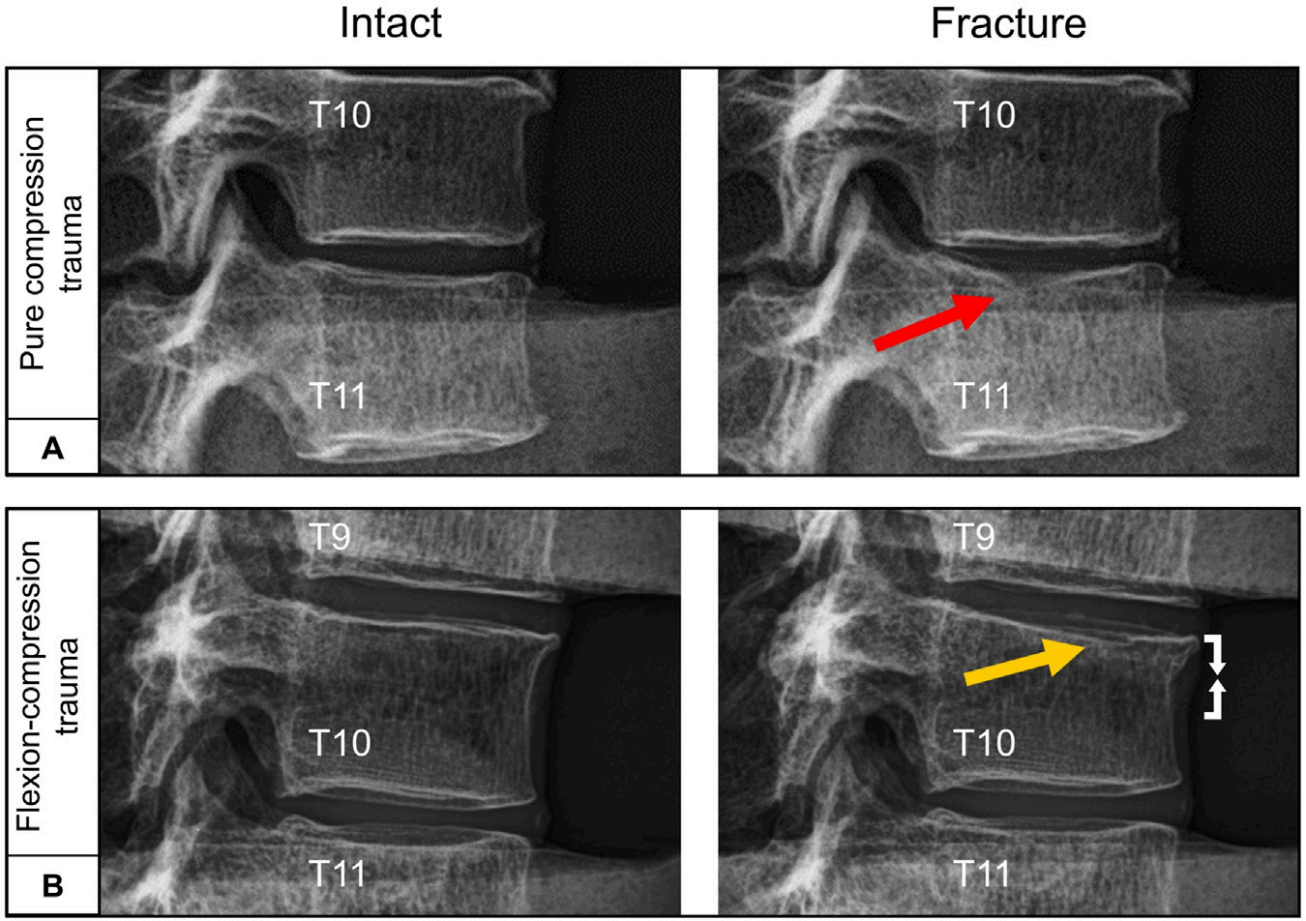
Exemplary lateral radiographs of the specimens (T9-T11) loaded by axial preload of 400 N before and after trauma: **(A)** Pure compression trauma primarily led to medial endplate fractures (red arrow). **(B)** Flexion-compression trauma predominantly resulted in combined medial endplate fractures (yellow arrow) and ventral compression injury (white arrows).

All specimens exhibited structural failure resulting from mechanical trauma simulation. The occurrence of traumatic injuries was observed in both trauma groups at a median fracture load of 5 kN (2.4–9.2 kN) within an exposure time of approximately 30 ms. During trauma simulation, median peak loads of 6.3 kN (4.0–10.7 kN) were recorded for pure compression trauma, while median peak loads of 5.2 kN (2.6–9.8 kN) were observed for flexion-compression trauma, with both trauma types showing load application within a timeframe of approximately 60 ms. Nine out of the twelve identified injury patterns were found to involve endplate structures. Pure compression trauma primarily resulted in isolated medial endplate fractures (n = 5), while flexion-compression trauma predominantly led to combined anterior endplate and superior vertebral compression injuries (n = 3) (Figure 3). In solely one specimen, pure compression trauma also produced an isolated vertebral compression injury (n = 1). Following flexion-compression trauma, pure superior vertebral compression injuries (n = 2) and one pure medial endplate fracture (n = 1) were additionally observed (Table 1). Regardless of the trauma type and morphological differences, however, all traumatic injuries were classified as AOSpine type A1 compression fractures (Schnake et al., 2017).

### 3.2 Instability analysis

#### 3.2.1 Flexibility analysis

**FIGURE 4:**
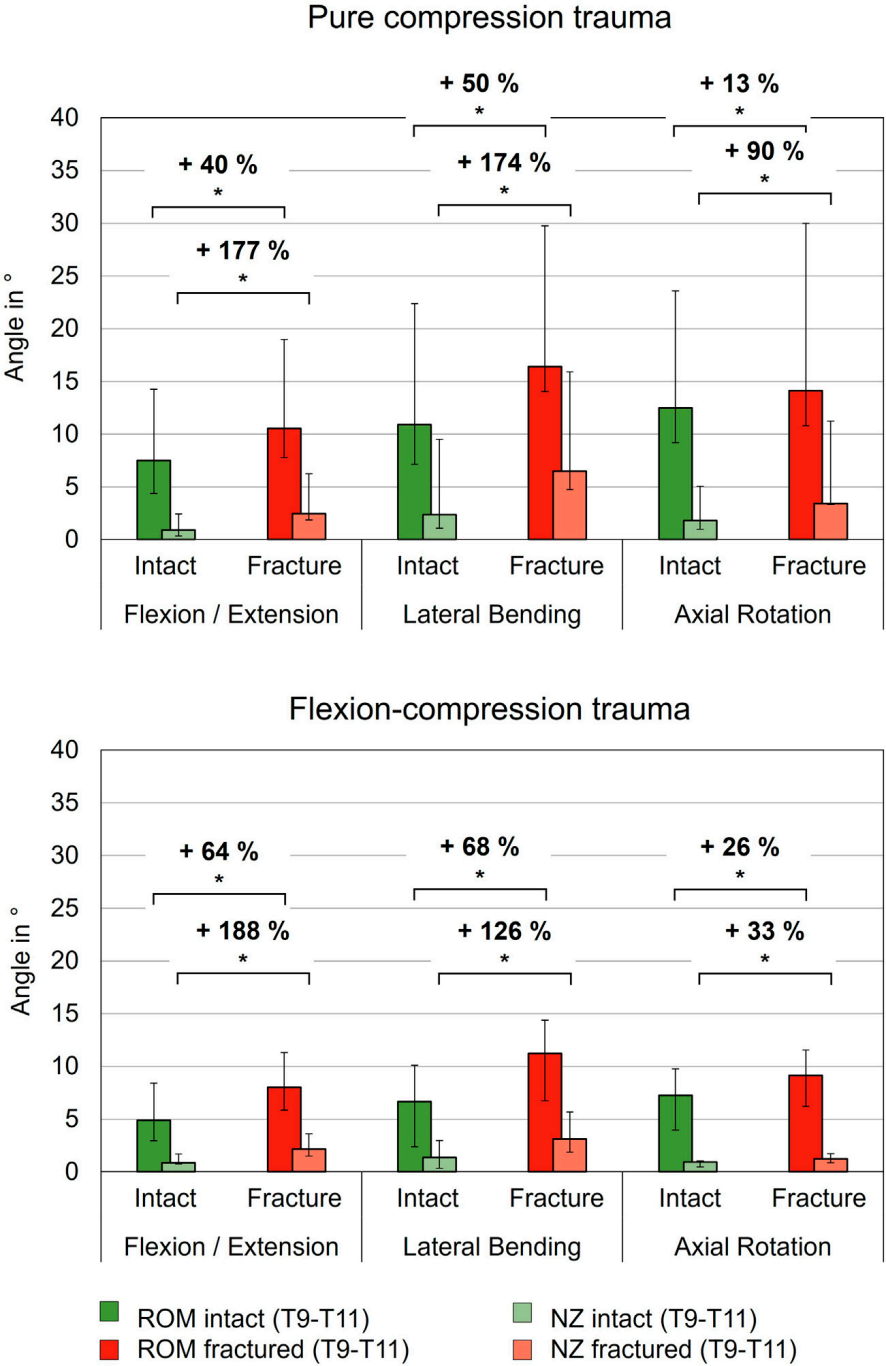
Range of motion (ROM) and neutral zone (NZ) of the specimens (T9-T11) before (Intact) and after trauma (Fracture) for pure compression and flexion-compression trauma, each presented as median with range (n = 6). Significant differences (p < 0.05) between the intact and post-traumatic condition are marked with an asterisk.

Pure compression trauma led to significantly increased range of motion and neutral zone in all three motion planes compared to the intact condition (Table 2; Figure 4). Range of motion increased by 40% (p = 0.01) in primary flexion/extension, by 50% (p = 0.01) in primary lateral bending, and by 13% in primary axial rotation (p = 0.01). Increases of the neutral zone values resulting from traumatic injury were generally higher than those observed in range of motion values. As a result of compression trauma, the neutral zone increased significantly, particularly in primary flexion/extension by 177% (p = 0.01) and in primary lateral bending by 174% (p = 0.01). During primary axial rotation, an increase of the neutral zone by 90% (p = 0.01) was observed. Flexion-compression trauma resulted in comparable increases in flexibility in terms of range of motion and neutral zone changes compared to the intact condition. Similarly, increases of the neutral zone were more pronounced compared to the range of motion. Following flexion-compression trauma, range of motion exhibited an increase by 64% (p = 0.01) in primary flexion/extension, by 68% (p = 0.01) in primary lateral bending, and by 26% (p = 0.01) in primary axial rotation. In contrast, in primary flexion/extension, the neutral zone increased almost three times as much as the range of motion by 188% (p = 0.01). In primary lateral bending, an increase of the neutral zone by 126% (p = 0.01) and in primary axial rotation by 33% (p = 0.01) were found.

**TABLE 2:** Range of Motion (ROM) and Neutral Zone (NZ) of the specimens (T9-T11) for intact and post-traumatic condition.

Motion plane	Flexibility parameter	Compression trauma		Flexion-compression trauma	
		Intact	Fracture	Intact	Fracture
		Median (Min, Max) in °	Median (Min, Max) in °	Median (Min, Max) in °	Median (Min, Max) in °
Flexion/Extension	ROM	7.5 (4.4, 14.3)	10.5 (7.8, 19.0) *	4.9 (2.9, 8.4)	8.0 (5.9, 11.3) *
	NZ	0.9 (0.4, 2.4)	2.5 (1.9, 6.2) *	0.7 (0.6, 1.6)	2.1 (1.5, 3.6) *
Lateral Bending	ROM	10.9 (7.1, 22.4)	16.4 (14.0, 29.8) *	6.7 (2.4, 10.1)	11.2 (6.8, 14.4) *
	NZ	2.4 (1.1, 9.5)	6.5 (4.7, 15.9) *	1.4 (0.3, 3.0)	3.1 (1.9, 5.7) *
Axial Rotation	ROM	12.5 (9.2, 23.6)	14.1 (10.8, 30.0) *	7.3 (4.0, 9.8)	9.1 (6.2, 11.5) *
	NZ	1.8 (1.0, 5.1)	3.4 (2.3, 11.2) *	0.9 (0.5, 1.0)	1.2 (0.9, 1.7) *

Min, Minimum; Max, Maximum; * = p < 0.05. (significant difference compared to the intact condition).

#### 3.2.2 Coupled motion analysis

While both trauma types resulted in significant flexibility increases in the primary motion directions, no significant changes in coupled rotations were found for secondary flexion/extension, secondary lateral bending, and secondary axial rotation after pure compression trauma (p > 0.05) and flexion-compression trauma (p > 0.05). In general, coupled rotations were almost nonexistent in the lower thoracic spine (Table 3).

**TABLE 3:** Coupled motions of the specimens (T9-T11) for intact and post-traumatic condition.

Trauma type	Testing condition	Coupled rotations	Flexion/Extension	Lateral bending	Axial rotation
			Median (Min, Max) in °	Median (Min, Max) in °	Median (Min, Max) in °
Compression trauma	Intact	Coupled flex./ext.	—	0.4 (0.1, 1.9)	0.6 (0.1, 1.7)
		Coupled lat. bend.	0.1 (0, 0.6)	—	0.8 (0.2, 2.3)
		Coupled ax. rot	0.3 (0, 2.2)	0.7 (0, 1.7)	—
	Fracture	Coupled flex./ext.	—	0.6 (0.2, 2.2)	0.6 (0.1, 2.2)
		Coupled lat. bend.	0.3 (0, 0.6)	—	0.8 (0.3, 2.7)
		Coupled ax. rot	0.4 (0.1, 3.3)	1.1 (0.1, 1.7)	—
Flexion-compression trauma	Intact	Coupled flex./ext.	—	0.5 (0.3, 0.7)	0.2 (0.1, 0.8)
		Coupled lat. bend.	0.3 (0, 0.7)	—	0.4 (0, 0.8)
		Coupled ax. rot	0.1 (0.1, 0.6)	0.4 (0, 0.7)	—
	Fracture	Coupled flex./ext.	—	0.6 (0.1, 1.2)	0.3 (0.1, 1.0)
		Coupled lat. bend.	0.5 (0.1, 1.0)	—	0.3 (0, 1.1)
		Coupled ax. rot	0.2 (0, 0.4)	0.3 (0.2, 0.8)	—

Min, Minimum; Max, Maximum.

**FIGURE 5:**
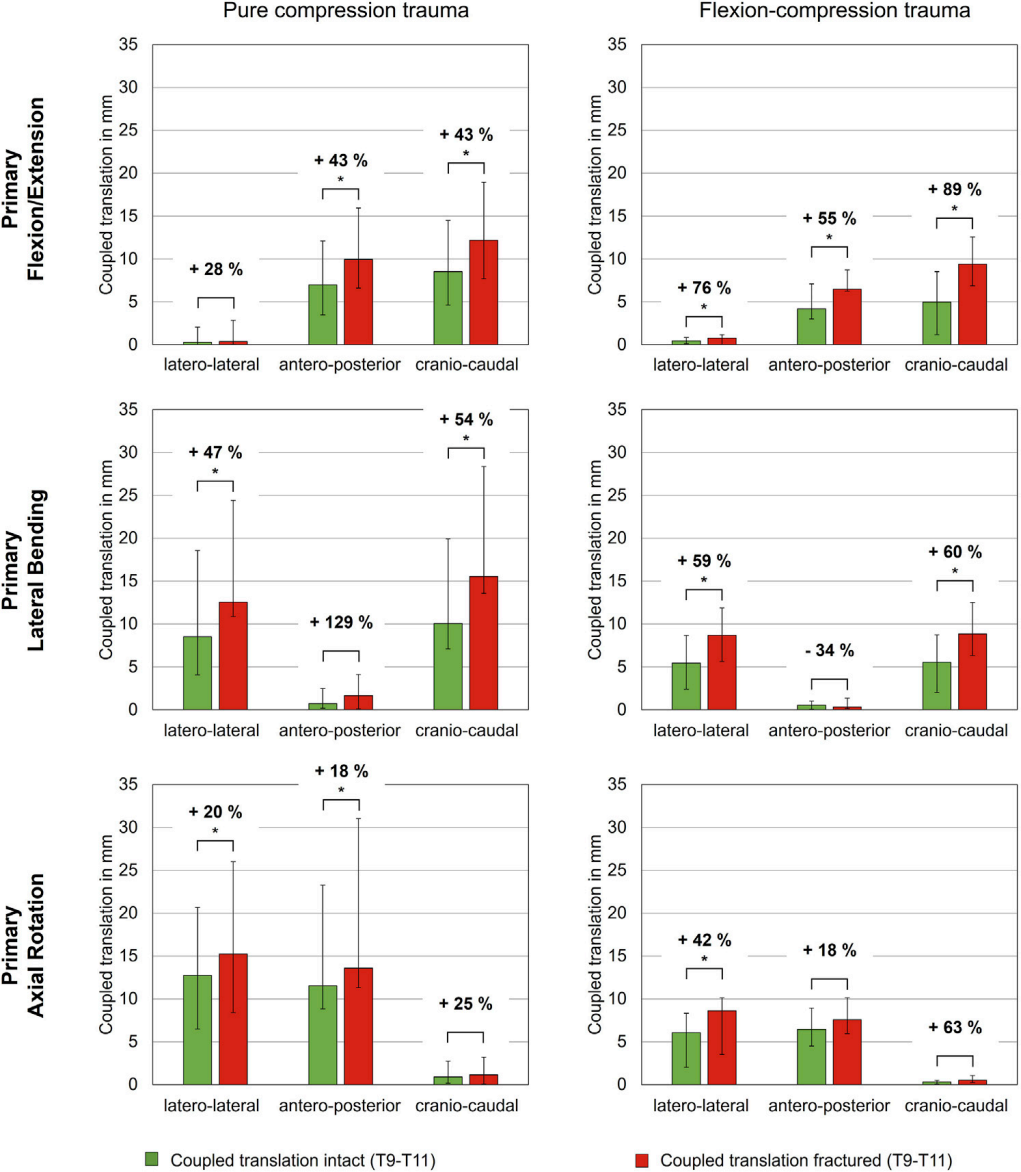
Coupled translatory flexibility during rotational motion along the latero-lateral, antero-posterior and cranio-caudal axes of intact and fractured specimens (T9-T10), each presented as median with range (n = 6). Significant differences (p < 0.05) between the intact and post-traumatic condition are marked with an asterisk.

In contrast, coupled translations during rotational motion increased significantly due to trauma. During flexion/extension, translational flexibility was detected primarily along the antero-posterior and cranio-caudal axes (Table 4; Figure 5). As a result of compression trauma, translation significantly increased by 43% along the antero-posterior axis (p = 0.01) and by 43% along the cranio-caudal axis (p = 0.01). Flexion-compression trauma led to even higher increases by 55% along the antero-posterior axis (p = 0.01) and by 89% along the cranio-caudal axis (p = 0.01). For lateral bending, coupled translations were found mainly along the latero-lateral and cranio-caudal axes. Again, post-traumatic increases were higher for flexion-compression trauma compared to pure compression trauma. After pure compression trauma, coupled translations increased significantly by 47% in latero-lateral direction (p = 0.01) and by 54% in cranio-caudal direction (p = 0.01), whereas after flexion-compression trauma, significant increases by 59% in latero-lateral direction (p = 0.01) and by 60% in cranio-caudal direction (p = 0.01) were detected. Coupled translations during axial rotation occurred mainly along the latero-lateral and anteroposterior axes. As with the other motion directions, flexion-compression trauma resulted generally in higher increases than pure compression trauma. Coupled translations along the latero-lateral axis increased significantly by 20% (p = 0.01) after pure compression trauma and by 42% (p = 0.01) after flexion-compression trauma. Along the antero-posterior axis, coupled translation increased by 18% for both trauma types, however, solely the increases in pure compression trauma were significant (p = 0.01).

**TABLE 4:** Coupled translatory motion of the specimens (T9-T11) for intact and post-traumatic condition.

Trauma type	Testing condition	Coupled translation	Flexion/Extension	Lateral bending	Axial rotation
			Median (Min, Max) in mm	Median (Min, Max) in mm	Median (Min, Max) in mm
Compression trauma	Intact	Antero-posterior	7.0 (3.5, 12.1)	0.7 (0.2, 2.5)	11.5 (8.8, 23.3)
		Latero-lateral	0.3 (0, 2.0)	8.5 (4.1, 18.6)	12.7 (6.5, 20.7)
		Cranio-caudal	8.5 (4.6, 14.5)	10.1 (7.1, 19.9)	0.9 (0.2, 2.8)
	Fracture	Antero-posterior	10.0 (6.6, 15.9) *	1.7 (0.1, 4.1)	13.6 (11.3, 31.0) *
		Latero-lateral	0.4 (0, 2.8)	12.5 (10.9, 24.4) *	15.3 (8.4, 26.0) *
		Cranio-caudal	12.2 (7.7, 19.0) *	15.5 (13.6, 28.4) *	1.1 (0.1, 3.2)
Flexion-compression trauma	Intact	Antero-posterior	4.2 (3.0, 7.1)	0.5 (0.1, 1.0)	6.5 (4.5, 8.9)
		Latero-lateral	0.4 (0.1, 0.8)	5.4 (2.4, 8.7)	6.1 (2.0, 8.3)
		Cranio-caudal	5.0 (1.1, 8.5)	5.5 (2.0, 8.7)	0.3 (0.1, 0.5)
	Fracture	Antero-posterior	6.5 (4.9, 8.7) *	0.3 (0.1, 1.3)	7.6 (6.0, 10.1)
		Latero-lateral	0.8 (0.2, 1.2) *	8.7 (5.6, 11.9) *	8.6 (3.5, 10.1) *
		Cranio-caudal	9.4 (3.7, 12.6) *	8.8 (6.3, 12.5) *	0.5 (0.2, 1.1)

Min, Minimum; Max, Maximum; * = p < 0.05. (significant difference compared to the intact condition).

#### 3.2.3 Translational flexibility analysis

**FIGURE 6:**
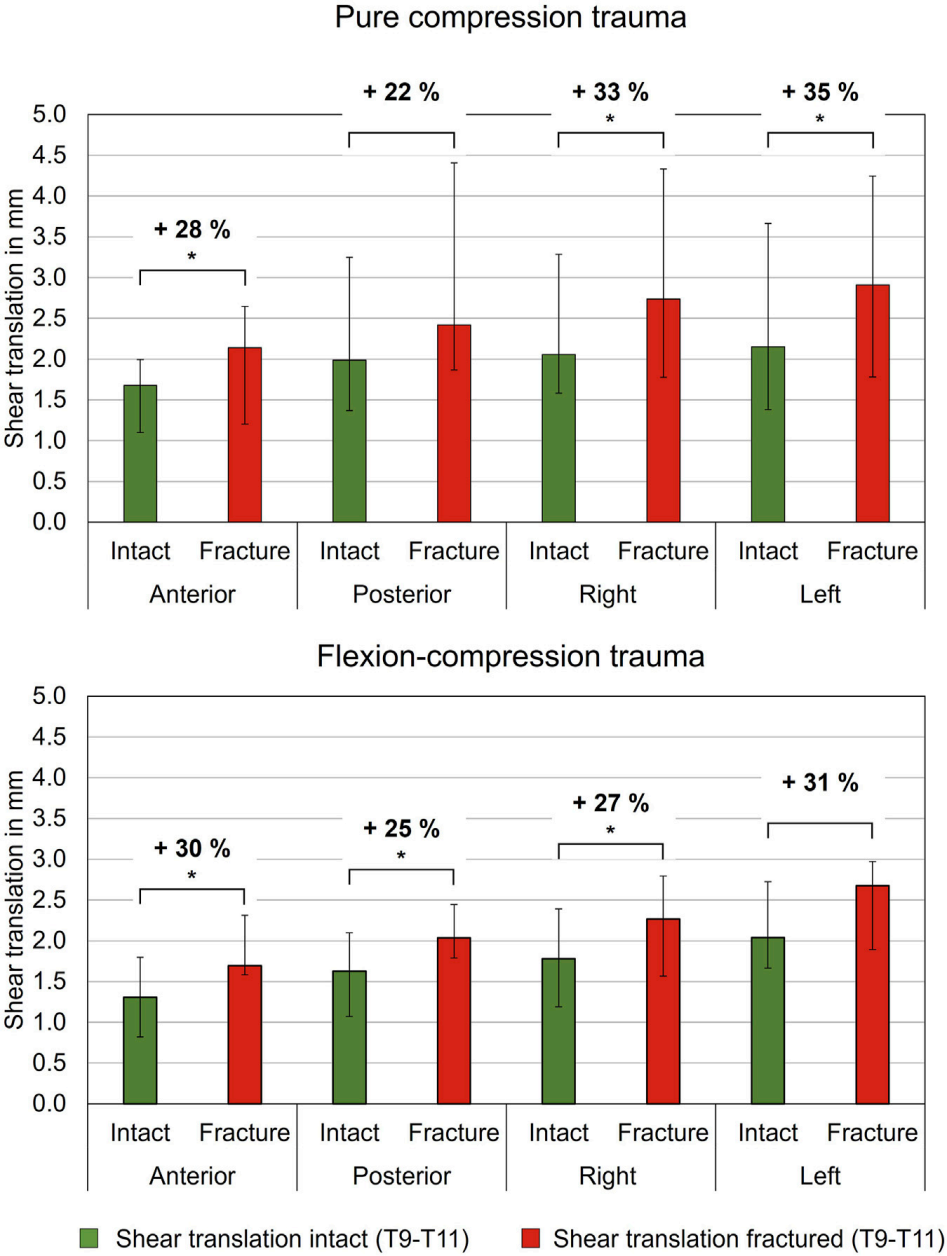
Translatory flexibility under shear loading of 100 N of intact and fractured specimens (T9-T10) after pure compression and flexion-compression trauma, each presented as median with range (n = 6). Significant differences (p < 0.05) between the intact and post-traumatic condition are marked with an asterisk.

Regarding shear translation, traumatic compression injuries led to an increase in translational flexibility (Table 5; Figure 6). After compression trauma, a significant increase by 28% (p = 0.01) in anterior direction was found under a shear load of 100 N. Similarly, lateral translation to the right increased by 33% (p = 0.01) and lateral translation to the left by 35% (p = 0.01). In contrast, no significant change in posterior translation was detected after pure compression trauma (p > 0.05). Increases in translational flexibility were also found in flexion-compression injuries. In anterior direction, a significant increase in translational flexibility by 30% (p = 0.01) was detected, while in posterior direction, an increase by 25% (p = 0.01) was evaluated. Furthermore, an increase in lateral translation to the right by 27% (p = 0.01) was measured. In contrast to pure compression trauma, changes in translational flexibility in lateral translation to the left were not statistically significant (p > 0.05).

**TABLE 5:** Shear translation of the specimens (T9-T11) for intact and post-traumatic condition in posterior, anterior and lateral directions due to a shear load of 100 N.

Translatory motion direction	Compression trauma		Flexion-compression trauma	
	Intact	Fracture	Intact	Fracture
	Median (Min, Max) in mm	Median (Min, Max) in mm	Median (Min, Max) in mm	Median (Min, Max) in mm
Anterior shear	1.7 (1.1, 2.0)	2.1 (1.2, 2.6) *	1.3 (0.8, 1.8)	1.7 (1.6, 2.3) *
Posterior shear	2.0 (1.4, 3.2)	2.4 (1.9, 4.4)	1.6 (1.1, 2.1)	2.0 (1.8, 2.4) *
Lateral right shear	2.1 (1.6, 3.3)	2.7 (1.8, 4.3) *	1.8 (1.2, 2.4)	2.3 (1.6, 2.8) *
Lateral left shear	2.2 (1.4, 3.7)	2.9 (1.8, 4.2) *	2.0 (1.7, 2.7)	2.7 (1.9, 3.0)

Min, Minimum; Max, Maximum; * = p < 0.05. (significant difference compared to the intact condition).

#### 3.2.4 Axial deformability analysis

**FIGURE 7:**
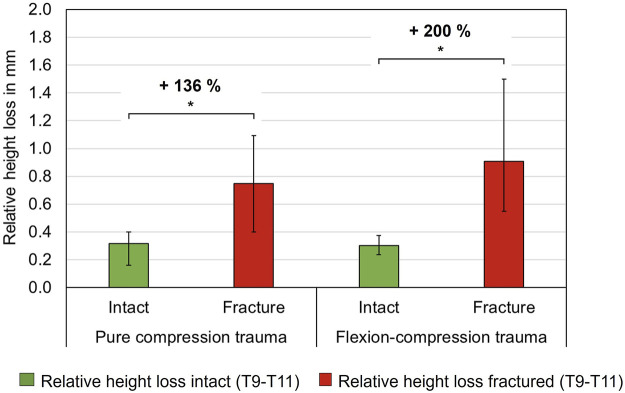
Axial deformability, so-called relative height loss, between loaded and unloaded condition (400 N vs. 0 N) of intact and fractured thoracic specimens (T9-T10), each presented as median with range (n = 6). Significant differences (p < 0.05) between the intact and post-traumatic condition are marked with an asterisk.

The simulated trauma led to a notable effect on relative axial deformability of the specimens. In both trauma groups, relative height loss between the unloaded (0 N) and the loaded condition (400 N) was significantly increased under compressive load after trauma simulation (p < 0.05). However, a more pronounced alteration in axial deformation was detected in flexion-compression trauma, with a median relative height loss increasing from 0.3 mm to 0.9 mm, representing a 200% increase (p = 0.01). In comparison, following pure compression trauma, median relative height loss increased significantly from 0.3 mm to 0.7 mm (p = 0.01), corresponding to an increase of 136% (Table 6; Figure 7).

**TABLE 6:** Axial deformability of the specimens (T9-T11) calculated as the difference between the unloaded and loaded state under an axial load of 400 N (relative height loss) for intact and post-traumatic condition.

Compression trauma		Flexion-compression trauma	
Intact	Fracture	Intact	Fracture
Median (Min, Max) in mm	Median (Min, Max) in mm	Median (Min, Max) in mm	Median (Min, Max) in mm
0.3 (0.2, 0.4)	0.7 (0.4, 1.1) *	0.3 (0.2, 0.4)	0.9 (0.5, 1.5) *

Min, Minimum; Max, Maximum; * = p < 0.05. (significant difference compared to the intact condition).

### 3.3 Influence of person-specific factors

#### 3.3.1 Age and sex vs. fracture load

No statistically significant correlation was found between donor age and fracture load for either pure compression (p > 0.05) or flexion-compression trauma (p > 0.05). Regarding donor sex, specimens from female donors tended to exhibit lower fracture loads than those from male donors. However, the two study groups comprised only two female and four male specimens each, respectively, which precluded statistical verification due to the limited sample sizes.

#### 3.3.2 Disc degeneration vs. fracture load

All intervertebral discs of the levels T9-T10 and T10-T11 of the tested specimens exhibited mild (Grade 1) to moderate (Grade 2) degree of disc degeneration according to the classification of Liebsch et al. (2022). When analyzing the relation between disc degeneration and fracture loads, there was a tendency for higher fracture loads in cases of more pronounced disc degeneration, especially in pure compression loading. However, no significant correlation was found between the degree of disc degeneration and fracture loads in either the pure compression (p > 0.05) or flexion-compression trauma group (p > 0.05).

#### 3.3.3 Bone mineral density vs. fracture load

**FIGURE 8:**
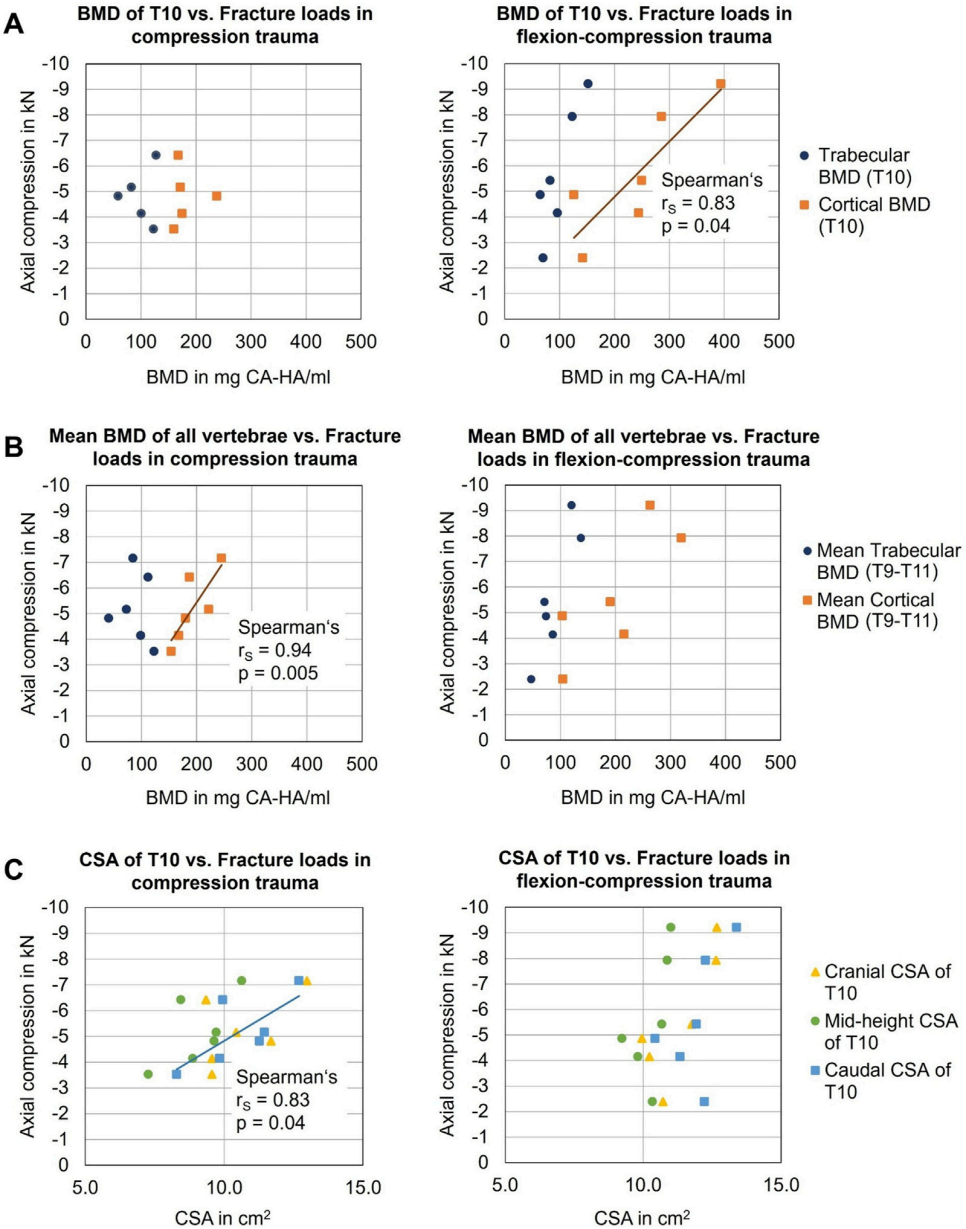
Analysis of the influences of person-specific factors on vertebral fragility: **(A)** Effects of bone mineral density (BMD) of target vertebra T10 on fracture loads in compression (n = 5) and flexion-compression trauma (n = 6). **(B)** Effects of mean BMD of all vertebrae (T9-T11) on fracture loads in compression (n = 6) and flexion-compression trauma (n = 6). **(C)** Effects of cross-sectional area (CSA) of T10 on fracture loads.

There was no statistically significant correlation between fracture load and cortical BMD of the T10 vertebra in case of pure compression trauma (p > 0.05). Similarly, no correlation was identified between trabecular BMD of T10 and fracture load (p > 0.05). However, when analyzing the mean cortical BMD of all vertebrae (T9-T11), a significant positive linear correlation was found between mean cortical BMD and fracture loads in pure compression trauma (p = 0.005) (Figure 8B) with a Spearman correlation coefficient r_s_ = 0.94 and a coefficient of determination *r* ^2^ = 0.88. For mean trabecular BMD, no correlation was found (p > 0.05).

For flexion-compression trauma, in contrast, a statistically significant positive linear correlation was found between fracture load and cortical BMD of T10 (p = 0.04) with a Spearman correlation coefficient r_s_ = 0.83 and a coefficient of determination *r* ^2^ = 0.69 (Figure 8A), suggesting that with higher BMD, higher loads were absorbed by the target vertebra T10. No significant correlation was detected between fracture loads and trabecular BMD of T10. Regarding mean cortical BMD and mean trabecular BMD of the entire specimens (T9-T11), no effects of required loads for fracture were found.

#### 3.3.4 Morphological parameters vs. fracture load

Analysis of the effect of vertebral body height on the fracture loads for both pure compression and flexion-compression trauma revealed no statistically significant correlation (p > 0.05, Table 7). Moreover, regarding the effect of intervertebral disc heights on fracture loads, no significant correlations were identified neither in pure compression nor in flexion-compression trauma (p > 0.05, Table 7).

**TABLE 7:** Morphological parameters of the specimens (T9-T10) including vertebral body height of T10, intervertebral disc height at levels T9-T10 and T10-T11, and cross-sectional area at cranial and caudal endplates as well as at mid-height of the target vertebra T10.

Morphological parameters	Compression trauma group	Flexion-compression trauma group
	Median (Min, Max)	Median (Min, Max)
aVBH T10 in mm	21.9 (16.8, 22.8)	20.4 (19.8, 22.3)
mVBH T10 in mm	21.1 (19.5, 22.9)	19.4 (17.2, 22.8)
pVBH T10 in mm	23.7 (22.7, 25.6)	22.9 (21.6, 26.2)
aIVDH T9-T10 in mm	5.0 (3.0, 6.5)	5.3 (2.8, 6.2)
mIVDH T9-T10 in mm	5.1 (3,9, 7.0)	6.0 (4.0, 7.3)
pIVDH T9-T10 in mm	3.6 (2.3, 4.4)	3.9 (3.2, 4.8)
aIVDH T10-T11 in mm	4.7 (3.7, 7.1)	6.8 (3.7, 8.9)
mIVDH T10-T11 in mm	5.4 (3.8, 6.6)	7.5 (5.8, 10.8)
pIVDH T10-T11 in mm	3.4 (2.5, 4.6)	4.0 (3.5, 5.2)
CSA of cranial endplate T10 in cm^2^	10.00 (9.35, 12.98)	11.24 (9.96, 12.67)
CSA of mid-height T10 in cm^2^	9.26 (7.28, 10.63)	10.51 (9.24, 11.01)
CSA of caudal endplate T10 in cm^2^	10.61 (8.30, 12.70) *	12.08 (10.44, 13.39)

aVBH, anterior vertebral body height; mVBH, medial VBH; pVBH, posterior VBH; aIVDH, anterior intervertebral disc height; mIVDH, medial IVDH; pIVDH, posterior IVDH; CSA, cross-sectional area; Min, Minimum; Max, Maximum; * = p < 0.05 (significant correlation between morphological parameter and fracture load).

The caudal cross-sectional area of the target vertebral body T10 significantly correlated with resulting fracture loads in pure compression trauma (p = 0.04, Table 7), exhibiting strong positive linear correlation with a Spearman correlation coefficient r_s_ = 0.83 and a coefficient of determination of *r* ^2^ = 0.69 (Figure 8C), which indicates that higher loads were required to fracture vertebrae with a larger cross-sectional area. No further correlations were identified between fracture loads and cranial and mid-height cross-sectional areas in pure compression trauma. In addition, no effects of cranial, caudal, and mid-height cross-sectional areas on the fracture loads were determined for flexion-compression trauma.

## 4 Discussion

The concept of spinal instability resulting from traumatic spinal injury remains a topic of ongoing debate due to the lack of clear definitions. Detailed understanding of spinal instability and injury mechanisms could enhance surgical treatment as well as the optimization of fixating spinal implants. Therefore, this study aimed to quantify three-dimensional multiparametric instability of compression and flexion-compression injuries in order to reveal the sensitivity of biomechanical instability measures and to identify potential effects of person-specific factors on vertebral fragility.

The findings of the present study indicate that even minor compression and flexion-compression injuries to the lower thoracic spine resulting from dynamic trauma simulation led to increases in flexibility, represented by several biomechanical parameters. In terms of bending flexibility, it was found that neutral zone exhibited the greatest increases with the most pronounced alterations in flexion/extension and lateral bending. A comparison of trauma types revealed that the neutral zone increased similarly in flexion/extension and lateral bending following pure compression trauma, whereas in case of flexion-compression trauma, the neutral zone exhibited higher increases in flexion/extension than in lateral bending. These discrepancies in resulting three-dimensional flexibility characteristics might be attributed to varying injury patterns caused by different trauma types. For instance, flexion-compression trauma resulted in combined endplate and ventral compression injuries, while pure compression trauma primarily caused isolated medial endplate fractures. Consequently, the more pronounced ventral vertebral body injury might explain why increases in the neutral zone during flexion/extension are more prevalent in flexion-compression trauma.

Overall, the findings that the neutral zone exhibits the highest increases following compression trauma align with previous works of White and Panjabi, who regarded the neutral zone as the most sensitive indicator for the onset and progression of spinal instability (White and Panjabi, 1990). Consistently, Panjabi et al. also identified highest reductions in the neutral zone with fixation and muscle force applications compared to range of motion changes, further demonstrating the sensitivity of the neutral zone with regard to spinal stability (Panjabi et al., 1994a). Despite these findings, the majority of subsequent *in vitro* studies investigating instability and surgical stabilization of traumatic spinal injuries have predominantly focused on examining changes in range of motion in response to spinal trauma (Liebsch and Wilke, 2022c; Greiner-Perth et al., 2024).

In the present study, analysis of the absolute range of motion data revealed that all generated minor thoracic compression injuries showed highest overall flexibility in lateral bending, independent of the trauma type. Focusing on the relative flexibility increases, it was found that the tested thoracic specimens experienced higher increases of range of motion in flexion/extension and lateral bending compared to axial rotation following both pure compression and flexion-compression trauma. This aligns with previous findings that injuries to the thoracic spine rather tend to be unstable in flexion/extension, while injuries to the thoracolumbar and lumbar spine are more likely to remain unstable in axial rotation (Greiner-Perth et al., 2024). However, when comparing different trauma types, increases in range of motion were slightly higher in cases of flexion-compression trauma. Again, this is most likely because flexion-compression trauma caused a more severe degree of spinal injury, involving both endplate and ventral compression fractures.

Previous research groups already reported that different fracture morphologies lead to varying residual biomechanical spinal instability (Oxland et al., 1991; Ching et al., 1995). In a porcine spinal compression fracture model, Oxland et al. found that posterior disc and endplate injuries result in increased flexibility in lateral bending (Oxland et al., 1991). In addition, posterior ligament or extension injuries led to pronounced instability in flexion, while anterior disc, endplate, and capsular ligament injuries were more related to rotational instability (Oxland et al., 1991). In the present study, the generated minor compression injuries were mainly characterized by medial endplate fractures, which, according to Oxland et al. (1991), suggest increased flexibility in lateral bending. Flexibility changes in terms of absolute range of motion in the simulated compression injuries therefore align with prior conclusions. However, it should be emphasized that translations of findings from porcine injury models to human spinal injuries should generally be regarded with caution due to anatomical differences.

In terms of coupled rotations, the present study did not detect any trauma-related changes. This may be attributed to the low severity of the simulated injuries. Generally, trauma-induced changes in coupled rotations have only been little investigated so far. Panjabi et al. are among the few authors who have reported on coupled motions (Panjabi et al., 1994b). In this study, also no changes in coupled rotations were found, even when analyzing more severe thoracolumbar burst fractures. However, when making direct comparisons, it must be highlighted to not only consider the differences in fracture severity but also the investigated spinal region, as the kinematics of the thoracic spine differ from those of the lumbar spine (Liebsch et al., 2018). Nonetheless, the findings obtained in both, the study of Panjabi et al. (1994b) and in the present study, indicate that coupled rotations are not affected by compression injuries in the thoracic and thoracolumbar spine.

To the authors’ knowledge, this study is the first to investigate the effect of spinal trauma on translational flexibility comprising coupled as well as shear translation. Translatory spinal flexibility has only scarcely been investigated so far (Liebsch and Wilke, 2022c), however, clinical evidence suggests a link between translational instability in the transverse plane and delayed or non-healing of spinal fractures (Panjabi et al., 1988). In terms of biomechanical stability, however, the analysis of coupled translations seems to be relevant, as the results of this study demonstrate that even minor compression injuries lead to moderate increases in coupled translatory flexibility. Similarly, these injuries led to increases in shear translations, though these increases were slightly less than those determined for coupled translations. When evaluating translatory instability in more detail, combined endplate and ventral compression injuries in the lower thoracic spine result in higher increases in latero-lateral translation compared to anterior-posterior translation. This might be because the posterior structures remain intact in these minor compression injuries, thereby limiting instability in the anterior-posterior direction. However, in order to develop treatment recommendations for restoring translational stability and to gain a more complete understanding of translational instability, further studies are required, particularly on more severe injury types and other spinal regions.

Regarding the analysis of instability, the present study detected the most significant increases in axial deformability under axial compressive loading of 400 N. A comparison of the resulting instability between different trauma types revealed that flexion-compression injuries exhibited higher increases in axial deformability compared to pure compression trauma. This can again be attributed to the more severe injury morphology. The increased deformability under compressive loading in general may be explained by the collapse of the cranial endplate and the subsequent penetration of the nucleus pulposus into the bony structure under axial compressive loading. In flexion-compression trauma, additional ventral compression injuries may further enhance axial deformability, making it even more pronounced.

In contrast to the outcomes of the present study, which demonstrated substantial increases in flexibility resulting from minor endplate injuries and combined endplate and vertebral compression injuries, previous research has shown contradictory results. To date, the study of Kifune et al. remains the sole investigation examining the instability resulting from endplate fractures (Kifune et al., 1995). However, they reported no significant changes in spinal motion characteristics, such as range of motion and neutral zone, following endplate fractures in the thoracolumbar spine. In their study, initial significant alterations in these parameters were solely detected in more severe fracture morphologies, beginning with wedge fractures. Similarly, other research groups have reported significant increases in flexibility in compression and wedge fractures in the thoracolumbar and lumbar spine (Kettler et al., 2006; Xu et al., 2006; Ruger and Schmoelz, 2009; Schmoelz et al., 2010; Disch and Schmoelz, 2014; Achatz et al., 2017). More detailed instability analyses of minor compression and endplate fractures, however, do currently not exist in literature. This lack of comprehensive studies underscores the necessity for further research to enhance the understanding of the biomechanical effects and fracture mechanisms associated with this common type of injury.

To better understand spinal injury mechanisms, the present study revealed that pure compression trauma predominantly resulted in isolated medial endplate fractures, whereas flexion-compression trauma led to combined medial endplate and superior ventral compression injuries of the vertebral body. The occurrence of medial endplate fractures under compression trauma can be attributed to the biomechanical properties of the intervertebral disc, functioning as a viscoelastic damping structure that distributes loads to adjacent vertebrae. Under pure axial loading conditions, the nucleus pulposus serves as the primary structure responsible for transmitting forces to the adjacent endplate. It is hypothesized that during dynamic impacts, the hydrated viscoelastic nucleus behaves like a rigid sphere, generating a concentrated force peak on the central endplate (Shirado et al., 1992), which may account for the frequent occurrence of medial endplate fractures under conditions of pure axial compression trauma (Magerl et al., 1994). Beyond the biomechanical behavior of the intervertebral disc, the alignment of the target vertebral body may also affect fracture localization. As the aim of this study was to simulate physiological load transmission, it was ensured that the load was centrally transferred to the T10 vertebra. Therefore, the embedding of the specimens was aligned horizontally to the endplates of T10 and during dynamic trauma simulation, the vertebra was centrally located in both the sagittal and frontal planes, potentially explaining the frequent occurrence of medial endplate fractures. The differing fracture severity observed for both trauma types can be attributed to the varied load distributions. In flexion-compression trauma, the anterior column predominantly bears the load, while the facet joints experience reduced loading. This results in higher stresses anteriorly, leading to failure in the ventral region of the vertebral body and subsequently causing a compression injury. Conversely, in pure compression trauma, the posterior column additionally serves as a load-bearing structure, distributing the load more effectively so that no additional compression injury occurs. Notably, previous research has demonstrated that the facet joints bear up to 33% of total axial forces, depending on the degree of extension (Nachemson, 1960). Although these different trauma mechanisms could explain different load distributions and thus different injury morphologies, no higher fracture loads were evaluated in pure axial compression trauma, although higher loads may have been transmitted via the facet joints. In this study, median fracture loads of 5 kN were determined across both trauma types. This finding aligns with previous findings of Kifune et al. (1995), who reported mean fracture loads of 4.8 kN in simulations of cranial endplate fractures in the thoracolumbar spine.

In addition to biomechanical instability parameters, this study aimed at investigating the effects of person-specific factors on vertebral fragility. Age, donor sex, and several morphological parameters such as vertebral body height and intervertebral disc height had no effects on required loads to initiate fracture development in this study. A significant correlation, however, was found between the cortical bone mineral density of the target vertebra T10 and the fracture loads in flexion-compression but not in pure compression trauma. One potential explanation for this is that in pure compression trauma, the load is distributed across the entire surface of the vertebra, the intervertebral disc, and the posterior structures, such as the facet joints. This load distribution might explain why the occurrence of fractures is more likely to correlate with the integrity of the posterior structures and morphological parameters, such as the cross-sectional area of the vertebral body. In fact, for pure compression trauma, this study found a significant correlation between the caudal cross-sectional area of T10 and fracture loads. This indicates that with a smaller cross-sectional area, lower loads were required to induce fractures, as a smaller cross-sectional area results in higher stresses within the vertebral body. In contrast, in flexion-compression trauma, the cross-sectional area of the target vertebral body had no effect on the fracture loads. Conversely, cortical bone mineral density appears to play a crucial role in flexion-compression trauma. A potential explanation may be the uneven load distribution due to flexion resulting in maximum stresses concentrated in the ventral region of the vertebral body. In addition, due to flexion, an increased load may be attributed to the annulus fibrosus of the intervertebral disc, leading to an uneven load distribution compared to the more uniform distribution through the nucleus pulposus during axial loading. Consequently, bone mineral density and cortical stiffness may have an increased influence on the vertebral collapse under these conditions in flexion-compression trauma. In contrast to the findings of this study, however, Shono et al. found a significant correlation between bone mineral density and failure loads in axial compression trauma (Shono et al., 1994). However, their analysis did not differentiate between cortical and trabecular bone mineral density and focused on generating burst fractures in thoracolumbar spine segments. Therefore, further research is needed to validate these findings as well as the previously stated hypotheses.

Regarding the effect of disc degeneration on fracture development, no correlation between the degree of disc degeneration and fracture loads were found. However, it should be noted that this study solely included thoracic spinal specimens with mild to moderate disc degeneration, not allowing investigations on healthy and severely degenerated discs, while it is known that already mild disc degeneration affects the biomechanics of the thoracic spine (Liebsch and Wilke, 2022b). Moreover, Shirado et al. identified that thoracolumbar spines with osteoporosis and discs that exhibited a higher degree of degeneration were less likely to suffer burst fractures (Shirado et al., 1992). One mechanical explanation could be that osteophyte formation and ossification, which accompany more severe degeneration, may lead to a different load distribution and higher cortical stiffness compared to healthy spines. These altered fracture mechanisms, combined with more prevalent osteoporosis in older patients, might result in a higher likelihood of suffering from compression fractures due to vertebral collapse rather than from burst fractures. This phenomenon was already pointed out by Roaf (1960) and Willen et al. (1984). Beyond the higher engagement in riskier activities among the younger population, this could also explain why burst fractures are more frequently observed in younger patients (Lenehan et al., 2009; Hasler et al., 2011; Tafida et al., 2018). However, direct translation of these findings to the present study may not be reasonable, as solely minor compression injuries in thoracic spines were simulated. Further studies with a larger specimen collective and simulations of more severe compression injuries, such as incomplete and complete burst fractures in both the thoracic and lumbar spine, are required to substantiate these hypotheses.

One limitation of this study represents its *in vitro* study design, as the loading conditions may not fully replicate the *in vivo* trauma situation. While *in vitro* trauma simulation and instability analysis were conducted on human specimens, providing better comparability to the *in vivo* situation than animal models, the exclusive use of human specimens cannot fully capture the spectrum of physiological responses. One reason is that the thoracic specimens used in this study lacked the stiffening effect of the rib cage, which was shown to significantly contribute to spinal stability (Liebsch et al., 2017; Liebsch and Wilke, 2020; Liebsch and Wilke 2022a). Moreover, the trauma response of the thoracic spine regarding post-traumatic flexibility changes was investigated exclusively on passive spinal structures, thereby neglecting the influence of active stabilizing factors, such as spinal muscles.

One of the primary challenges in simulating spinal trauma is accurately assessing the loads to which the spine is subjected *in vivo* during traumatic events, which ultimately result in specific fracture morphologies. While trauma protocols utilizing drop towers have been widely accepted as the standard methodology for generating traumatic spinal compression injuries (Panjabi et al., 1994b; Panjabi et al., 1998), they are constrained by limitations such as estimating the required load to induce specific injury morphologies or relying on incremental approaches that mimic unphysiological fracture progression rather than a single traumatic event. Hence, the *in vitro* protocol for injury simulation developed in the present study is based on a displacement-controlled approach using a material testing machine, which was assumed to provide a higher degree of standardization in generating specific injury morphologies compared to a force-controlled method.

Estimating the velocity and the direction at which loads impact the spine during a traumatic event represents an additional challenge. While the precise duration of a traumatic impact acting on passive spinal structures remains unclear in the existing literature, in the present study, it was assumed that the maximum impact occurs faster than the response time of spinal muscles. Given that paraspinal muscles exhibit reaction times ranging from 60 to 100 ms (Olive et al., 2019), the present study aimed to simulate a traumatic impact inducing maximum load exposure within a timeframe of less than 100 ms. To facilitate rapid trauma simulation, the novel *in vitro* protocol developed in the present study was derived from the methodology of Hartensuer et al. (2012), which was recognized as the fastest established protocol in previous literature for simulating traumatic spinal injuries in a materials testing machine. Application of this protocol, which employed compression at a velocity of 300 mm/s, resulted in median fracture loads of 5 kN observed within 30 ms, while median peak loads of 6.3 kN and 5.2 kN were recorded within 60 ms across both trauma groups. In contrast, Panjabi et al. (1998) reported maximum loads up to 7.0 kN occurring within approximately 10 ms using a drop tower setup, highlighting comparable magnitudes of impact forces and timeframes between the two methodologies. However, as *in vivo* dynamic force impacts are presumed to be attenuated by anatomical structures and surrounding soft tissues before reaching the passive spinal structures, the traumatic impact applied in the present study may even provide a closer approximation to *in vivo* conditions.

Another limitation of this *in vitro* trauma model is represented by the inherent boundary conditions. The complete fixation of the specimens during trauma application may have introduced non-physiological constraining forces that could affect the natural behavior of the spine during traumatic impacts. Although it was assumed that potential constraining forces would be absorbed by the deformation of the intervertebral discs and care was taken during mounting to keep these forces as low as possible, future studies should further optimize the experimental setup to minimize potential distortions.

In terms of identifying risk factors for vertebral fragility, it should be noted that this study only involved a small group of specimens. Therefore, conclusions drawn from the analysis of person-specific parameters that affect the spinal response to traumatic events should be interpreted with caution. In particular, the investigation of the effects of donor sex and disc degeneration requires larger sample sizes to gain more profound insights into their impact on fracture generation, as the present study included a limited number of two female and four male donors per trauma group, and no specimens with absent or severe disc degeneration were examined. Expanding the sample size in future research would also strengthen findings related to bone mineral density and morphological parameters influencing vertebral fragility. Nonetheless, the correlation analyses performed in the present study remain valuable as they indicate trends regarding the effects of person-specific factors on vertebral fragility in spinal compression trauma and as they highlight parameters that warrant further investigation to enhance the understanding of underlying injury mechanisms.

A distinctive feature of the present study was that traumatic spinal fractures were induced by means of a purely mechanical trauma simulation, without the use of artificial defects created by resection. For the first time, mild compression fractures, especially endplate fractures, were systematically simulated in the lower thoracic spine following a single impact approach. Additionally, this study is the first to conduct a multiparametric instability analysis, providing a detailed insight into the specific three-dimensional instability characteristics of these simulated minor compression injuries in the lower thoracic spine. Nonetheless, to thoroughly understand biomechanical spinal instability associated with specific fracture types, further studies that also focus on more severe fracture patterns and which include different spinal regions are required. These limitations highlight the need for ongoing research to refine current experimental models and thereby enhance the applicability of experimental results to clinical scenarios.

## 5 Conclusion

The findings of the present study demonstrate that minor compression injuries to the lower thoracic spine can be effectively generated through pure mechanical trauma simulation without the application of artificial defects. Moreover, the findings indicate that even minor injury morphologies can result in significant increases of spinal flexibility. The most relevant instability measures for minor compression and flexion-compression injuries of the lower thoracic spine were found to include relative axial deformability under compressive loading as well as neutral zone (NZ) in flexion/extension and lateral bending, followed by coupled translations and alterations in range of motion (ROM). Conversely, only minor increases in shear translation and no significant alterations of coupled rotations were identified. Regarding risk factors associated with vertebral fragility, this study demonstrated that the cortical bone mineral density of the target vertebra T10 significantly affects fracture loads in flexion-compression trauma, but not in pure compression trauma. Conversely, the cross-sectional area of the target vertebra has a significant impact on fracture loads in pure compression trauma, but not in flexion-compression trauma. Thus, it can be concluded that risk factors for traumatic spinal fractures vary depending on trauma mechanisms.

Nevertheless, in order to achieve a comprehensive understanding of the underlying injury mechanisms for various types of traumatic spinal injuries and the associated biomechanical spinal instability, further studies are essential. Future studies should continue to perform multiparametric analysis of spinal instability in compression fractures, focusing on potential differences regarding spinal levels. Furthermore, there is a need for more detailed investigation of the biomechanical instability of more severe fracture morphologies, including burst fractures, hyperflexion, and extension injuries, in order to determine specific three-dimensional instability characteristics. This will contribute to a more comprehensive understanding of the biomechanical aspects of spinal instability associated with specific injury types in distinct spinal regions resulting from trauma, which could be used to optimize current treatment recommendations by integrating valid instability criteria into current treatment guidelines. By establishing more precise criteria for the assessment and surgical management of spinal injuries, treatment protocols could be refined to reduce complications associated with residual spinal instability and thereby improve patient outcomes.

## Data Availability

All relevant data are included in the article, further inquiries can be directed to the corresponding author.
